# Gap junction protein beta 4 plays an important role in cardiac function in humans, rodents, and zebrafish

**DOI:** 10.1371/journal.pone.0240129

**Published:** 2020-10-13

**Authors:** Ryuji Okamoto, Itaru Goto, Yuhei Nishimura, Issei Kobayashi, Ryotaro Hashizume, Yoshinori Yoshida, Rie Ito, Yuhko Kobayashi, Misato Nishikawa, Yusuf Ali, Shunsuke Saito, Toshio Tanaka, Yoshiki Sawa, Masaaki Ito, Kaoru Dohi

**Affiliations:** 1 Department of Cardiology and Nephrology, Mie University Graduate School of Medicine, Tsu, Mie, Japan; 2 Department of Molecular and Cellular Pharmacology, Pharmacogenomics and Pharmacoinformatics, Mie University Graduate School of Medicine, Tsu, Mie, Japan; 3 Core-Lab, Graduate School of Regional Innovation Studies, Mie University, Tsu, Mie, Japan; 4 Department of Pathology and Matrix Biology, Mie University Graduate School of Medicine, Tsu, Mie, Japan; 5 Department of Cell Growth and Differentiation, Center for iPS Cell Research and Application, Kyoto University, Sakyo-ku, Kyoto, Japan; 6 Department of Cardiovascular Surgery, Osaka University Graduate School of Medicine, Suita, Osaka, Japan; Scuola Superiore Sant'Anna, ITALY

## Abstract

**Aims:**

GJB4 encodes a transmembrane connexin protein (Cx30.3) that is a component of gap junctions. This study investigated whether GJB4 plays an important role in human heart disease and function.

**Methods and results:**

We examined a patient and her older brother who both presented with complicated severe hypertrophic cardiomyopathy (HCM) and whose parents are healthy married cousins. The gene exome analysis showed 340 single nucleotide polymorphisms (SNPs) that caused amino acid changes for which the patient was homozygous and both parents were heterozygous. After excluding all known common (>10%) SNP gene mutations, the gene for GJB4 was the only identified gene that is possibly associated with cardiac muscle. The resultant E204A substitution exists in the 4th transmembrane domain. GJB4-E204A impaired the binding with gap junction protein A1 (GJA1) compared with GJB4-WT. The expression of GJB4 was induced in rat disease models of left and right ventricle hypertrophy and mouse disease models of adriamycin-induced cardiomyopathy and myocardial infarction, while it was not detected at all in control. An immunohistochemical study was performed for autopsied human hearts and the explanted heart of the patient. GJB4 was expressed and colocalized with GJA1 in intercalated discs in human diseased hearts, which was extensively enhanced in the explanted heart of the patient. The abnormal expression and localization of GJB4 were observed in beating spheres of patient’s induced pluripotent stem cell (iPSC)-derived cardiomyocytes (CMs). We generated knockout zebrafish of *GJB4* by CRISPR/Cas9 and the endodiastolic volume and the ventricular ejection fraction were significantly lower in GJB4-deficient than in wild-type zebrafish at five days post-fertilization.

**Conclusions:**

These results indicate both that GJB4 is defined as a new connexin in diseased hearts, of which mutation can cause a familial form of HCM, and that GJB4 may be a new target for the treatment of cardiac hypertrophy and dysfunction.

## Introduction

The dilated phase of hypertrophic cardiomyopathy (d-HCM) is a primary underlying diagnosis for adult heart transplant recipients around the world [1] and the second leading cause of heart transplantation in Japan [2]. HCM is the most frequent cardiomyopathy and is known as a sarcomere abnormality disease, because gene mutation analysis in familial HCM has shown the existence of mutations in sarcomere-associated genes, including myosin heavy chain (*MYH7*), myosin-binding protein C (*MYBPC3*), troponin T (*TNNT2*), and troponin I (*TNNI3*). However, these reported mutations in cardiac sarcomere proteins account only for 60% of cases of HCM [3]. Therefore, it is anticipated that additional gene mutations cause HCM.

Myocardial disarray is accompanied by the disorganized gap junction formation and thought to be a cause of arrhythmia [4]. Gap junctions are intercellular conduits for small molecules made up by gap junction proteins (GJPs). So far, 21 GJP genes have been identified [5], and the human heart has been reported to express four kinds of GJPs: gap junction protein A1 (GJA1), also known as connexin (Cx) 43; gap junction protein A4 (GJA4), also known as Cx37 [6]; and gap junction protein A5 (GJA5), also known as Cx40 and gap junction protein gamma 1 (GJC1), or as Cx45 [7]. GJA1 is the most abundant in the heart and is expressed in both atria and ventricles, while GJA5 is expressed in atria and the conduction system. GJC1 is located exclusively in the conduction system, while GJA4 is expressed in the endothelial gap junctions in vessels. Disturbances of GJP complexes may account for abnormalities of impulse propagation, contributing to arrhythmia and myocardial mechanical insufficiency [7]. Gap junction protein beta 4 (GJB4), also known as Cx30.3, is normally expressed in skin with GJA1. Although it remains unknown whether GJB4 plays an important role in cardiac physiology and function, GJB4 is transiently but strongly expressed when stem cells differentiate into cardiomyocytes [8]. Importantly, GJB4 can work as a normal functional channel like GJA1 [5].

We have encountered a patient with severe familial hypertrophic cardiomyopathy in Mie Prefecture, Japan. Her older brother died suddenly when he was a boy, and the autopsy showed hypertrophic cardiomyopathy. She was diagnosed with HCM, and during the 20-year follow-up, HCM progressed to d-HCM and needed cardiac resynchronization therapy with defibrillator (CRT-D). The patient received a left ventricular-assist device implant (HeartMate II). At age 41, she has undergone heart transplantation with no complications. Their parents are married parallel cousins, whose mothers are sisters. Their parents and grandparents were free of cardiac hypertrophy and failure. Therefore, this familial hypertrophic cardiomyopathy is considered to be an autosomal recessive disease. We performed genome analysis using next-generation sequencing, and a mutation in *GJB4* was found as a candidate gene. We evaluated the expression and the localization of GJB4 in patient’s iPSC-derived cardiomyocytes (CMs) and diseased heart from mouse, rat and human, the loss of function of GJB4 in zebrafish by CRISPR/Cas9 and the prevalence of this mutation in patients with cardiomyopathy.

## Methods

### Patient with HCM, parents, and other patients

For all participants, informed written consent was obtained, and the study protocol was approved by the Mie Graduate University School of Medicine (No. 1408, 1484 and No. 2158).

### Mutational analysis with cDNA

The patient was screened for mutations using myocardial biopsy samples for cDNAs of four myofilament genes, including *MYH7*, *MYBPC3*, *TNNT2* and *TNNI3*. Direct DNA sequencing was used.

### Exome sequencing and bioinformatics analysis

Exome sequencing and variant annotation were performed using the Core-Lab, Graduate School of Regional Innovation Studies in Mie University. Exome sequencing was performed on three persons, the patient and her parents (Table 1 and S1 Table). After exon capture was performed, exon libraries were sequenced using Ion AmpliSeq technology (Life Technologies, Carlsbad, CA, USA) with an Ion Torrent Personal Genome Machine (PGM) platform. The reads were aligned to human genome 37 (hg19). Allele frequencies were generated for known variants in the 1000 Genomes databases (http://www.1000genomes.org. Accessed November 22, 2013) and GnomAD (https://gnomad.broadinstitute.org/ Accessed April 1, 2017). Classification and annotation of genetic variants was accomplished using CLC Genomics Workbench 6.0.4 (CLC bio. Inc., Aarhus, Denmark).

**Table 1:** Summary of SNP analysis in the affected individual.

Items	
Total observed SNPs	70,647
Filtering	
SNPs causing amino acid change	9,520
Accumulative homozygous	340
≤10% in dbSNP	34

The exome library used for whole exome sequencing (WES) was prepared using an Ion Torrent AmpliSeq Exome RDY kit (Thermo Fisher Scientific, Inc. Waltham, MA, USA) in accordance with the manufacturer’s recommended protocol. Fifty nanograms of high-quality genomic DNA was used for target amplification under the following conditions: 99°C for 2 min, followed by 10 cycles at 99°C for 15 s and 60°C for 16 min, and a final hold at 10°C. Incorporated primer sequences were partially digested using a proprietary method. Ion Torrent Proton adapters were ligated to the amplicons at 22°C for 30 min followed by 72°C for 10 min, and the library was purified with Agencourt Ampure XT beads (Beckman Coulter Inc., Brea, CA, USA). Libraries were quantified using quantitative polymerase chain reaction (qPCR), and DNA (8 pM) was sequenced using a semiconductor DNA sequencer (Ion Torrent Proton Sequencer, Thermo Fisher Scientific) according to the manufacturer’s protocol.

Data from the Proton runs were initially processed using Ion Torrent platform-specific pipeline software, Torrent Suite v4.0 (Thermo Fisher Scientific) to generate sequence reads, trim adapter sequences, and filter and remove poor signal-profile reads. Initial variant calling from the Ion AmpliSeq™ sequencing data was generated using Torrent Suite with a plug-in ‘variant caller’ program. To eliminate erroneous base calling, three filtering steps were used to generate final variant calling. The first filter was set at an average depth of total coverage of >50, a per variant coverage of >15, and P<0.01. The second filter was employed by CLC Genomics Workbench version 6.0.4 (Qiagen, Hilden, Germany), as well as by filtering out possible strand-specific errors (i.e., a mutation was detected only in one, but not both, strands of DNA).

### Animal models

The rats and mice were maintained in the Mie University animal facilities. The Standing Committee on Animals at Mie University approved all protocols pertaining to experimentation with animals (No. 23–34 and No. 24–25). Mie University Institutional Animal Care and Use Committee guidelines state that no approval is required for experiments using zebrafish. Mice and rats were euthanized and sacrificed with carbon dioxide at determined time or just after showing any severe heart failure symptoms including tachypnea, reduced mobility and abnormal behaviors. No animal died before meeting criteria for euthanasia. The animal health and the animal behavior were monitored twice a day. Efforts to minimize suffering and distress were performed with the use of analgesics.

### Angiotensin II (AngII)-induced left ventricular hypertrophy in rats

Male Sprague-Dawley rats (8 weeks old, 200–250 g) from Japan SLC (Hamamatsu, Japan) were treated with angiotensin II at 60 ng/kg/min and 200 ng/kg/min for one week by osmotic pumps (ALZET 2002; ALZA Corp., Palo Alto, CA, USA) as previously reported [9]. Hearts were collected for western blot analysis after mice were sacrificed.

### Right ventricular hypertrophy in rats

Male Sprague-Dawley rats (6 weeks old, 160–180 g) from Japan SLC were given a single subcutaneous injection of SU5416 (20 mg/kg) and exposed to hypoxia (10% O_2_) for 3 weeks to induce pulmonary artery hypertension (PAH), then returned to normoxia as previously reported [10]. Rats were sacrificed at 9–10 weeks after SU5416 injection, and hearts were collected for analysis.

### Adriamycin (ADM)-induced cardiomyopathy in mice

Eight-week-old male C57BL/6J mice (SLC) received adriamycin (ADM, Wako, Osaka, Japan) intraperitoneally at 4 mg/kg every other day for a total of 2 weeks as previously reported [11]. Surviving mice were sacrificed 2 weeks after cessation of ADM treatment.

### Myocardial infarction in mice

Myocardial infarction (MI) by ligation of the left anterior descending coronary artery was studied in 10- to 12-week-old male C57BL/6J mice (SLC), as described previously [12]. Anesthesia was induced with 3.0% isoflurane inhalation with 100% oxygen, followed by intubation and respiratory support with a rodent volume-controlled mechanical ventilator (VentElite 55–7040, Harvard Apparatus, Holliston, MA, USA) at a tidal volume of 3 ml and 80 breaths/min. A 4th left thoracotomy was performed to expose the heart, following by ligating the proximal left anterior descending coronary artery with a 12–0 polypropylene suture. Myocardial ischemia was confirmed by decreased movement in the left ventricle free wall and regional cyanosis. Three days after induction of MI, the mice were sacrificed and their hearts were collected for western blot analysis.

### Constructs of expression vectors

Mammalian expression vectors encoding wild-type and mutant GJB4 and wild-type GJA1 (pCMV-FLAG-GJB4 and pCMV-HA-GJA1, respectively) were prepared by subcloning full-length human GJB4 and GJA1 cDNAs, respectively, into pCMV FLAG tagged vector (pCMVTag4A™; Stratagene, San Diego, CA, USA) and pCMV-HA vector (Sigma, St. Louis, MO, USA). The primers used were: for GJB4, 5′- GTC GAC TCA TGA ACT GGG CAT TTC TGC AGG GC-3′ and 5′- GTC GAC TTA TGG ATA CCC ACC TGC ATC CAC-3′ (forward and reverse primers, respectively; underlined residues show the *Sal*I sites, respectively); for GJA1, 5′- GTC GAC TCA TGG GTG ACT GGA GCG-3′ and 5′- GTC GAC CTA GAT CTC CAG GTC ATC AGG-3′ (forward and reverse primers, respectively; underlined residues show the *Sal*I sites, respectively).

The conditions used for the PCR amplification were 94°C for 15 s, 58°C for 30 s, and 68°C for 1 min for 30 cycles using KOD plus™ DNA polymerase (Toyobo Osaka, Japan). The fragments were subcloned into pCR2.1 TOPO vector, followed by digestion with *Sal*I. Each fragment was sequenced to confirm that the PCR-amplified cDNA was identical to the original sequence.

### Immunoprecipitation of GJA1 and GJB4 in Cos7 cells

Cos7 cells were grown by standard procedure [13]. Subconfluent Cos7 cells were transfected with HA- or FLAG-tagged DNA constructs (1 μg/ml) with Lipofectamine 3000 (Invitrogen, Carlsbad, CA, USA). Immunoprecipitation of GJA1 from Cos7 cells was as follows: Cos7 cells were homogenized with modified RIPA lysis buffer (50 mM Tris-HCl, pH 7.4, 1% NP40, 150 mM NaCl, 0.05% sodium deoxycholate, 10% glycerol, 10 mM Na_4_P_2_O_7_, 10 mM NaF, 2 mM Na_3_VO_4_, and 1× protease inhibitor mixture). The supernatant after centrifugation at 19,000 × g. for 10 min was collected and incubated with anti-HA antibody (Santa Cruz Biotechnology, Dallas, TX, USA, rabbit polyclonal) for 3 h, followed by additional incubation with protein A-Sepharose (GE Healthcare, Little Chalfont, UK) for 30 min at 4°C. The immunoprecipitates were washed three times with buffer (20 mM Tris-HCl, pH 7.4, 500 mM NaCl, 0.1% NP40, and 0.05% sodium deoxycholate) and followed by an additional wash three times with the same buffer without NaCl. The immunoprecipitated proteins were eluted with Laemmli SDS sample buffer and immediately boiled for 5 min.

### Western blotting

Proteins extracted from tissues or cells were separated on SDS-PAGE, transferred to PVDF membranes, and probed with each antibody as described previously [13]. Anti-GJB4 (rabbit, ab199178), anti-GJA1 (rabbit, ab11370; mouse, ab79010) and anti-β-actin (rabbit, ab8227) antibodies were purchased from Abcam (Cambridge, UK). Anti-FLAG antibody was obtained from Sigma (mouse, F1804). Anti-HA antibodies were obtained from Santa Cruz (rabbit, sc-805) and Wako (mouse, 018–21884).

### Immunostaining of GJB4 and GJA1 in autopsied human normal and diseased hearts, the explanted heart of the patient and the sphere of iPSC-induced cardiomyocytes

Immunohistochemical staining was performed on normal, diseased autopsied d-HCM and hypertensive human hearts, the explanted heart of the patient undergoing cardiac transplantation for advanced d-HCM and spheres of iPSC-induced cardiomyocyotes using a labeled streptavidin-biotin method (DAKO Corp., Santa Clara, CA, USA). Left ventricular free wall samples from the human heart were formalin-fixed and paraffin embedded. Deparaffinized 3-μm-thick sections were heated for 5 min at 100°C in a pressure cooker to reactivate the antigen, then treated with 0.3% H_2_O_2_ in methanol for 30 min to abolish endogenous peroxidase activity. Sections were blocked with 10% goat serum in PBS, incubated with the primary antibody (anti-GJB4 or anti-GJA1 rabbit antibody (Abcam)) overnight at 4°C, washed, covered with second-step biotinylated antibody for 30 min, and incubated with peroxidase-labeled streptavidin for 30 min. After washing, sections were then incubated with 0.05% diaminobenzidine/0.15% H_2_O_2_ and counterstained with 10% hematoxylin. Image acquisition was done with a BZ-X700 microscope (Keyence, Osaka, Japan).

### Generation of patient’s specific induced pluripotent stem cell, differentiation into cardiomyocytes, purification and analysis of cardiomyocyte-derived spheres

In vitro iPSC cardiac differentiation. We developed iPSC from patient’s skin fibroblasts using the four Yamanaka factors and SNL feeder cells according to the same protocol as previously shown [14] (https://www.cira.kyoto-u.ac.jp/j/research/img/protocol/hipsprotocolv2_090304.pdf) and used the human iPSC line 201B7 as control. We differentiated iPSC into cardiomyocyte using embryoid body in high efficiency ≈80% at day 20 after the induction using our established cardiac differentiation protocol [15, 16]. In brief, undifferentiated iPSCs were detached into single cells with Accumax (Innovative Cell Technologies, Inc, San Diego, CA). The cells were placed into a low-attachment 96-well dish to form to form embryonic bodies (EBs) after resuspension in StemPro34 medium supplemented with 2 mM L-glutamine, 50 μg/ml ascorbic acid, 39μg/ml monothioglycerol, 150 μg/ml transferrin, 10 μM Y-27632 (WAKO), 2 ng/ml human recombinant BMP4 (R&D Systems, Minneapolis, MN) and 0.5% matrigel. Twenty-four hours later (day 1), the same volume of StemPro 34 medium, which included 12 ng/ml human activin A (R&D Systems) (final concentration 6ng/ml), 18 ng/ ml BMP4 (final concentration 10ng/ml), and 10 ng/ml bFGF (R&D Systems) (final concentration 5ng/ml), were added into the wells. On day 3, EBs were isolated into single cells by 5-min incubation with Accumax. The dissociated cells were suspended in differentiation media including 10 ng/ml VEGF (R&D Systems) and 1 μM IWP-3, a Wnt inhibitor (Stemgent, Cambridge, MA) and then put in each well (approximately 30,000 to 50,000 cells/well) to form aggregates for 4 days. On day 7, the media were replaced with StemPro 34 media supplemented with 2 mM L-glutamine, 50 μg/ml ascorbic acid, 39μg/ml monothioglycerol, 150 μg/ml transferrin, 5 ng/ml VEGF. We changed the culture media every 2–3 days for the maintenance of iPSC-CMs. On day 20, we recorded video images of iPSC-CMs and fixed with 5% paraformaldehyde and proceed them into immune staining of GJB4 and GJA1.

### Development of GJB4-knockout zebrafish by CRISPR/Cas9

We obtained Tg (myl7:mRFP) zebrafish, which express mRFP under the control of the myosin light chain 7 (myl7) promoter, a gene selectively expressed in cardiomyocytes [17], from the National BioResource Project Zebrafish (Saitama, Japan). Zebrafish were bred and maintained according to previously described methods [18]. Briefly, zebrafish were raised at 28.5°C ± 0.5°C with a 14-h/10-h light/dark cycle. Embryos were obtained by natural mating and cultured in 0.3× Danieau’s solution (19.3 mM NaCl, 0.23 mM KCl, 0.13 mM MgSO_4_, 0.2 mM Ca(NO_3_)_2_, 1.7 mM HEPES, pH 7.2) until 5 five days post-fertilization (dpf), at which time they were used for *in vivo* imaging analyses or were processed for qPCR.

Knockout of GJB4 (Cx34.4 in zebrafish, the ortholog of human GJB4) was performed by the ready-to-use CRISPR/Cas9 method [19]. CRISPR RNA (crRNA) targeting a 5’- AGTCGCGGCTGAAAAAGTGT -3’ sequence in the GJB4 gene and transactivating crRNA (tracrRNA) were obtained from FASMAC (Kanagawa, Japan). Recombinant Cas9 protein was obtained from Toolgen (Seoul, South Korea). In brief, crRNA, tracrRNA, and Cas9 protein were dissolved in sterilized water at concentrations of 250, 1000, and 1000 ng/μl, respectively, and stored at -80°C until required. For microinjection, crRNA, tracrRNA, Cas9 protein, and a lissamine-labeled control morpholino with no known target gene (Gene Tools, Philomath, OR, USA) were mixed in Yamamoto’s Ringer’s solution (0.75% NaCl, 0.02% KCl, 0.02% CaCl_2_, 0.002% NaHCO_3_) to final concentrations of 100 ng/μl, 100 ng/μl, 400 ng/μl, and 50 nM, respectively. The solution was injected into 1-4-cell-stage zebrafish embryos derived from the Tg (myl7:mRFP) line. At 1 dpf, embryos exhibiting bright lissamine fluorescence were selected and maintained until 5 dpf. At 5 dpf, selected zebrafish were used for *in vivo* imaging of cardiac ventricles. After completion of *in vivo* imaging experiments, genomic DNA was extracted from the zebrafish by incubation in 50 μl of lysis buffer (10 mM Tris-HCl, pH 8.0, 0.1 mM EDTA, 0.2% Triton X-100, 200 μg/ml proteinase K) at 55°C overnight, followed by incubation at 99°C for 10 min. The solution was then placed at 4°C and used as the template for PCR. To detect the crRNA-induced mutations, a short fragment of the GJB4 gene encompassing the crRNA target sites was amplified from genomic DNA using GJB4_gF1 and GJB4_gR1 primers and QuickTaq (Toyobo). PCR cycling conditions were: 94°C for 2 min followed by 40 cycles of 94°C for 30 s, 60°C for 30 s, and 68°C for 30 s. The PCR products were electrophoresed on 10% polyacrylamide gel (Wako) and visualized by ethidium bromide staining. The crRNA, tracrRNA, and PCR primer sequences are shown in (S2 Table).

### *In vivo* imaging of the zebrafish heart

GJB4-KO zebrafish at 5 dpf were transferred onto glass slides. A few drops of 3% low-melting point agarose were laid over the living larvae, which were immediately placed on their backs. The ventricles of the embedded larvae were observed using an epifluorescence microscope (SMZ25; Nikon, Tokyo, Japan) with RFP filters, and images were recorded at 100 frames/s for 10 s. Quantitative assessment of cardiac function was performed using ImageJ and Volocity software (Perkin Elmer, Cambridge, MA, USA). Briefly, time-lapse images were processed using the Fast Fourier Transform package in ImageJ to reduce the background noise, and the long and short diastolic and systolic diameters of the ventricles were measured using Volocity. The endodiastolic volume (EDV) and endosystolic volume (ESV) were calculated using the diameters. The ejection fraction (EF) was calculated from the EDV and ESV.

### Statistics

All data are expressed as the mean ± standard error of the mean. Statistical analysis was performed by unpaired Student's *t* test or one-way analysis of variance followed by Tukey’s *post hoc* test. A value of P < 0.05 was considered statistically significant.

## Results

### *GJB4* is a candidate gene causing autosomal recessive familial HCM

We interviewed the patient and her parents and referred to their medical records. Her older brother, who was eleven years old, died suddenly and the autopsy showed hypertrophic cardiomyopathy. At that time, the patient was eight years old and was diagnosed with HCM by echocardiogram. She has been followed in the local hospital for about thirty years. When she was 31 years old, she developed d-HCM and was admitted due to heart failure. At 37, she developed syncope due to non-sustained VT and was transferred to our hospital for implantation of a cardiac resynchronization therapy-defibrillator (CRT-D). At 42, she has successfully undergone heart transplantation two years after she received implantation of Heartmate. cDNA analysis from her cardiac samples showed that sequences of *MYH7*, *MYBPC3*, *TNNT2*, and *TNNI3* were normal.

**Fig 1:**
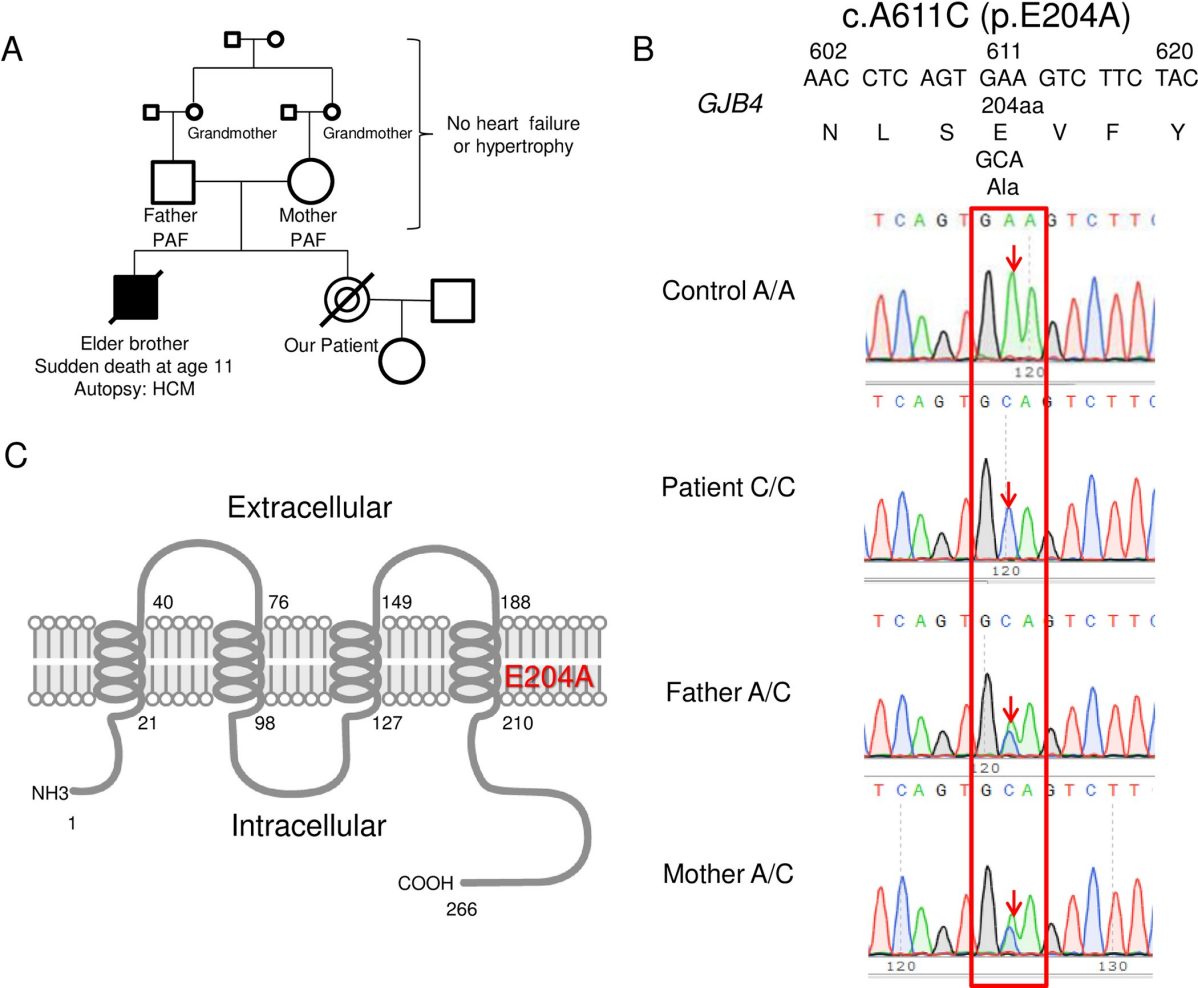
Familial hypertrophic cardiomyopathy due to mutations in *GJB4*. (A) Pedigree of the affected family. Squares denote male family members, circles denote female members, and diagonal lines indicate diseased members. PAF, paroxysmal atrial fibrillation. HCM, hypertrophic cardiomyopathy. (B) Partial sequence electropherogram of *GJB4* with the nucleotide substitution in the three affected members analyzed. The heterozygous mutation c.A611C causes a missense substitution of p.E204A. (C) Schematic representation of the GJB4 protein with the position of the mutation identified in patients with HCM.

The patient’s parents are married parallel cousins whose mothers are sisters (Fig 1A). Their parents and grandparents had no history of heart failure. Parents recently developed paroxysmal atrial fibrillation without cardiac hypertrophy. Therefore, this familial hypertrophic cardiomyopathy is considered as an autosomal recessive disease (Fig 1A). Gene exome analysis showed 340 single nucleotide polymorphisms (SNPs) that caused amino acid changes for which the patient was homozygous and both parents were heterozygous (Table 1). After excluding all known common (>10%) SNP gene mutations in the SNP Database (dbSNP), 34 genes remained, as shown in Table 2.

**Table 2:** Polymorphic nonsynonymous or splicing site variants in candidate genes from exome data.

No.	Gene	RefSeq	Chromosome	Nucleotide change	Amino acid change	SNP quality	Read depth	Mutant allele no.	dbSNP140	Mutant allele frequency/GnomAD_exomes	Mutant allele frequency/1000 Genomics	Polyphen2 score
1	HMGB4	NM_145205	Ch1	c.413T>C	p.Val121Ala	24.51	138	138	N/A	0	0	N/A
**2**	**GJB4**	**NM_153212**	**Ch1**	**c.611A>C**	**p.Glu204Ala**	**25.92**	**87**	**86**	**rs3738346**	**0.039**	**0.101**	**1.000**
3	UROD	NM_000374	Ch1	c.254A>C	p.Gly85Val	25.43	60	60	rs14186676	N/A	0.009	N/A
4	C1orf112	NM_018186	Ch1	c.1441A>C	p.Ala481Ser	26.33	486	486	rs2272920	0.073	0.050	0.999
5	SCYL3	NM_020423	Ch1	c.1628G>C	p.Gly543Ala	26.00	473	470	rs12143301	N/A	0.047	0.004
6	FAM179A	NM_199280.2	Ch2	c.2521C>T	p.Arg724Cys	25.25	217	217	rs60403047	0.091	0.094	0.002
7	TTL	NM_153712	Ch2	c.317A>G	p.Lys106Arg	25.53	89	89	rs141830950	0.0002	0.001	N/A
8	PCDP1	NM_001271049	Ch2	c.2014A>T	p.Thr672Ser	26.03	91	90	rs186209494	0.0001	0.001	N/A
9	DNAH12	NM_178504	Ch3	c.547C>A	p.Leu183Ile	25.98	41	41	rs79825658	0.025	0.035	N/A
10	GP9	NM_000174	Ch3	c.466G>A	p.Ala156Thr	25.52	52	52	rs3796130	0.047	0.068	0.688
11	TRIM39-RPP21	NM_001199119	Ch6	c.1492C>A	p.Gln498Lys	25.19	26	26	rs35287137	N/A	N/A	N/A
12	HLA-C	NM_001243042	Ch6	c.526G>A	p.Ala176Thr	25.44	123	118	rs41552817	N/A	0.027	N/A
13	NFKBIE	NM_004556	Ch6	c.581T>C	p.Val194Ala	21.67	83	83	rs2233434	0.049	0.074	0.000
14	NFKBIE	NM_004556	Ch6	c.524C>T	p.Pro175Leu	25.08	225	223	rs2233433	0.046	0.062	N/A
15	MICAL1	NM_001159291	Ch6	c.2014GC>AA	p.Ala672Lys	24.64	66	66	rs35260632	N/A	0	N/A
16	PLEKHG1	NM_001029884	Ch6	c.2143G>A	p.Ala715Thr	26.34	170	170	rs79596384	N/A	0.036	N/A
17	PPP1R3A	NM_002711	Ch7	c.2649G>T	p.Arg883Ser	26.66	125	125	rs1800000	0.032	0.048	0.005
18	LZTS1	NM_021020	Ch8	c.1423C>G	p.Leu475Val	23.28	29	29	rs723874	N/A	0.086	0.440
19	C8orf86	NM_207412	Ch8	c.651T>G	p.Ile217Met	26.48	31	31	rs74846385	0.020	0.039	N/A
20	SPATA6L	NM_001039395	Ch8	c.745C>G	p.His249Asp	25.89	22	19	rs41302077	0.047	0.083	N/A
21	FREM1	NM_144966	Ch9	c.4421C>T	p.Thr1474Ile	24.83	188	186	rs41265306	N/A	0.084	N/A
22	FAM154A	NM_153707	Ch9	c.799C>T	p.Pro267Ser	26.61	309	308	rs113182590	0.007	0.016	N/A
23	PRRC2B	NM_013318	Ch9	c.3335T>C	p.Leu1112Pro	23.31	52	49	rs74696187	0.005	0.006	N/A
24	LRRC32	NM_001128922	Ch11	c.1219G>A	p.Ala407Thr	24.97	269	264	rs79525962	0.026	0.045	N/A
25	CCDC83	NM_001286159	Ch11	c.145A>G	p.Thr49Ala	24.60	315	312	rs12362209	0.062	0.044	0.032
26	APLP2	NM_001142276	Ch11	c.1858G>A	p.Asp620Asn	25.71	122	121	rs3740881	0.018	0.052	0.014
27	PTPRQ	NM_001145026	Ch12	c.3125A>G	p.Asp1042Gly	24.88	83	75	rs190166486	0.0002	0.001	N/A
28	SACS	NM_001278055	Ch13	c.12598A>G	p.Ile4200Val	25.12	303	300	rs200939906	N/A	0.001	N/A
29	GOLGA8A	NM_001023567	Ch15	c.1463C>T	p.Ala488Val	24.45	22	22	rs142225671	N/A	0	N/A
30	EFCAB13	NM_001195192	Ch17	c.547A>G	p.Ile183Val	26.48	48	48	rs55853213	0.062	0.063	0.071
31	ELP2	NM_018255	Ch18	c.2384A>G	p.Glu795Gly	23.76	58	58	rs12607773	0.012	0.038	0.001
32	SIGLEC11	NM_001135163	Ch19	c.1510G>A	p.Asp504Asn	24.43	128	128	rs45438992	0.053	0.077	N/A
33	ADAMTS1	NM_006988	Ch21	c.2108G>A	p.Arg703His	25.52	128	128	rs56251528	0.056	0.061	N/A
34	XKR3	NM_175878	Ch22	c.695C>T	p.Pro232Leu	24.77	43	43	rs114989947	N/A	0.034	N/A

N/A, not available.

The gene for gap junction protein beta 4 (*GJB4*) was the only identified gene that is possibly associated with cardiac muscle. The PolyPhen-2 scores of mutations in *GJB4* were 1.000 (Table 2), and the identified missense mutation was a 611 A>C nucleotide transition (Fig 1B). The resultant E204A substitution replaces a polar carboxylic glutamate with a nonpolar alanine (Fig 1B). GJB4 has four transmembrane domains, two extracellular loops, a cytoplasmic loop, and cytoplasmic N- and C-termini (Fig 1C). E204A exist in the 4th transmembrane domain (Fig 1C).

### GJB4 is upregulated in rat models of cardiac hypertrophy in left and right ventricles and in mouse models of cardiomyopathy and myocardial infarction

**Fig 2:**
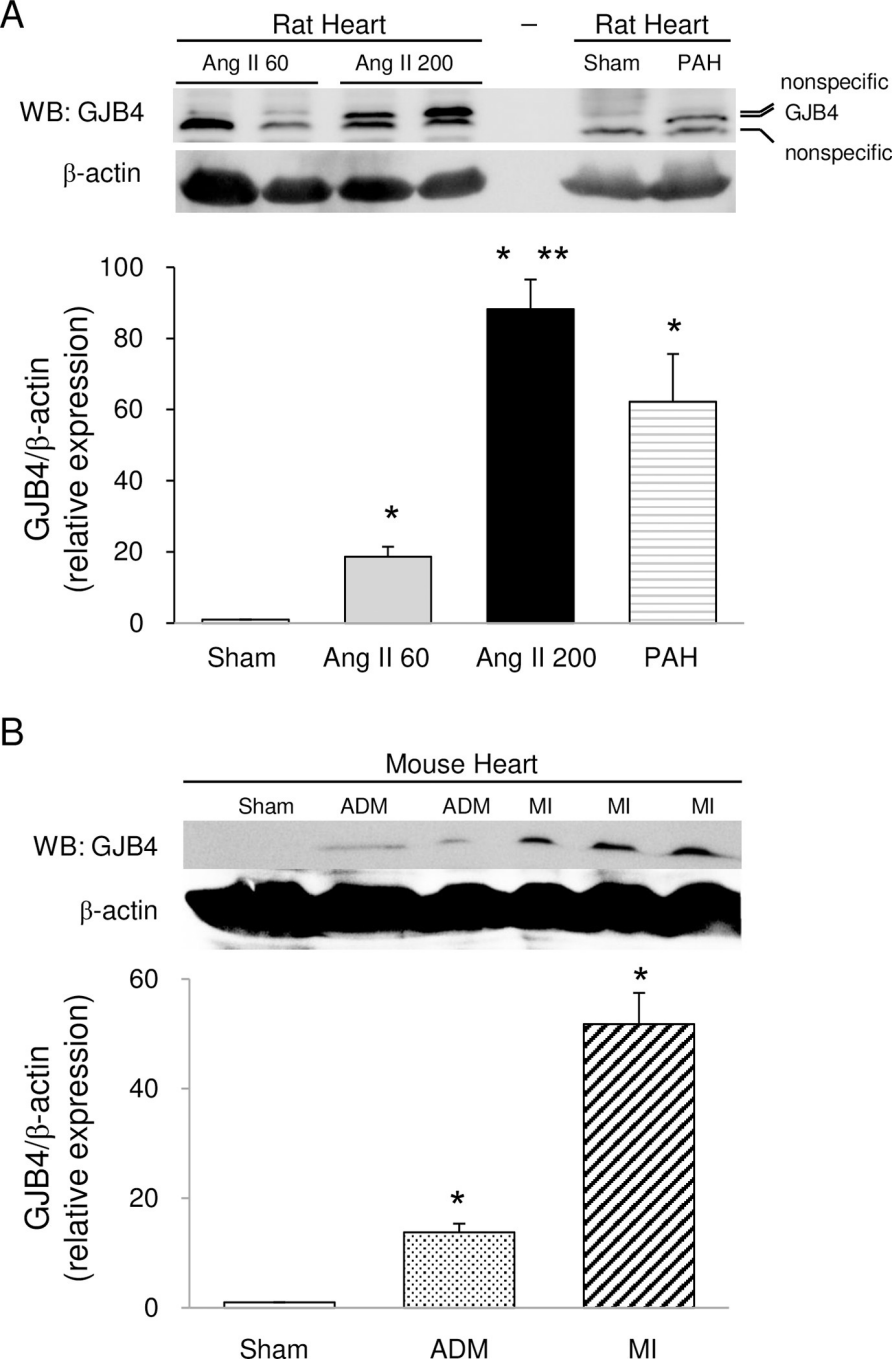
GJB4 is expressed only in diseased hearts. Expression of GJB4 as shown by western blot analysis. (A) Immunoblot showing expression of GJB4 and β-actin in hearts from rats treated with Ang II 60 (60 ng/kg/min) and Ang II 200 (200 ng/kg/min) for one week, sham-operated rats, and SU5416-induced pulmonary arterial hypertension (PAH) rats (n = 4/group). *P<0.01 vs. saline-treated rats (Sham). **P<0.05 vs. Ang II 60-treated rats (Sham). (B) Immunoblot showing expression of GJB4 and β-actin in hearts from mouse models of adriamycin (ADM)-induced cardiomyopathy and myocardial infarction (MI) (n = 4/group). *P<0.01 vs. saline-treated mice (Sham).

To investigate changes in the expression of GJB4 in diseased hearts, we examined the expression of GJB4 in hearts from rats treated with angiotensin II or SU5416 under hypoxia to develop cardiac hypertrophy and in mice treated with adriamycin or left coronary artery ligation to develop heart failure. Interestingly GJB4 was expressed in hearts from rat hypertrophy models and from mouse heart failure models, while it was not detected at all in sham-operated rats and mice (Fig 2).

### Impaired interaction of GJB4-E204A with GJA1 compared with GJB4-WT

**Fig 3:**
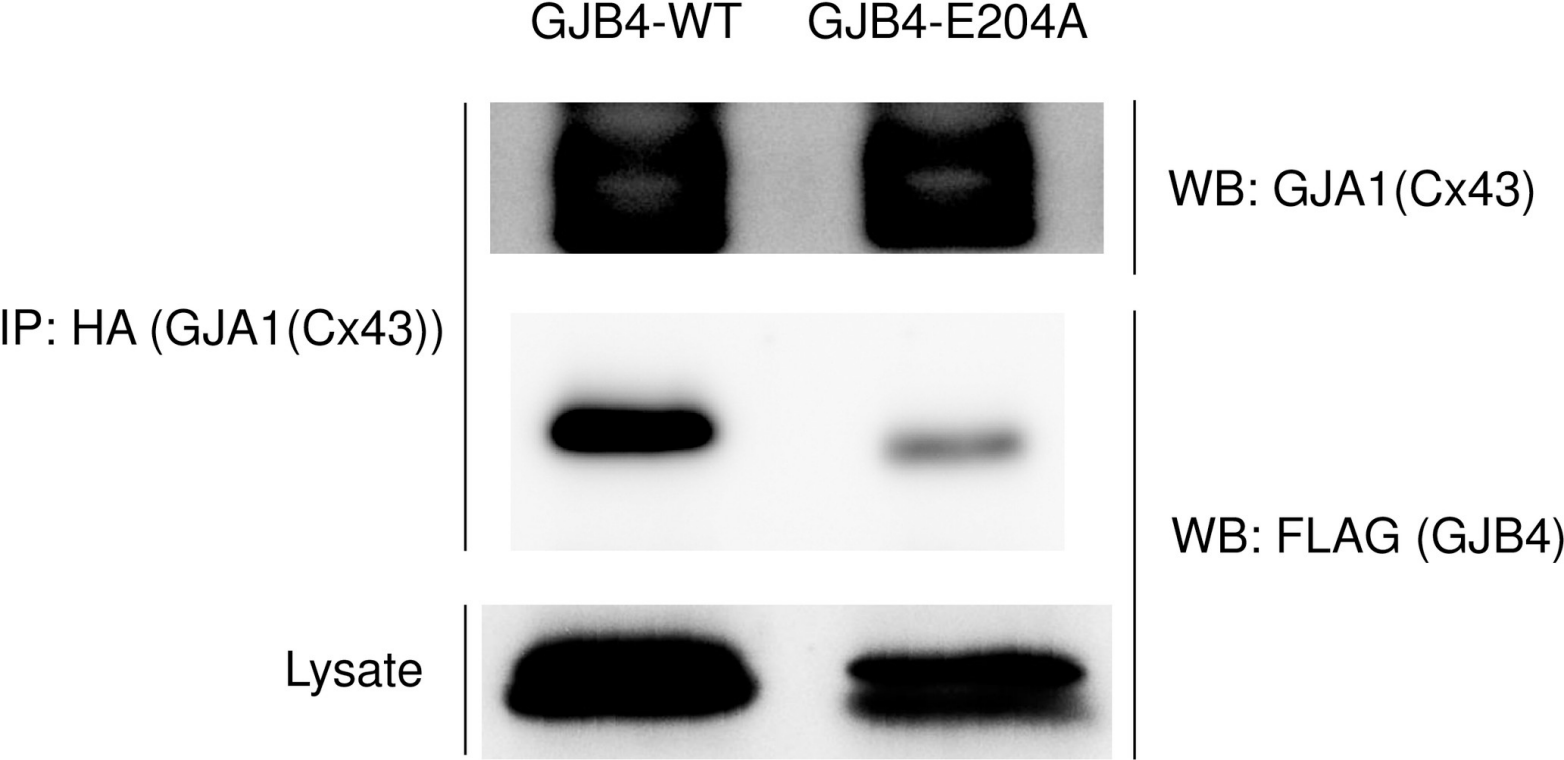
Weaker interaction of GJB4-E204A with GJA1 than GJB4-WT. Coimmunoprecipitation of GJB4 and GJA1 in Cos7 cells overexpressing FLAG-GJB4-E204A or FLAG-GJB4-WT and HA-GJA1 (n = 3).

There are functional redundancy and compensation for hexamers of subunits among member of GJPs. Indeed functional replacement of one GJP by another is common and GJB4 can work as GJA1 [5]. Next, to investigate the interaction of GJA1 with GJB4, FLAG-tagged GJB4-E204A or GJB4-WT vectors were co-transfected with HA-tagged GJA1 vector into Cos7 cells, followed by immunoprecipitation with anti-HA antibody. The band of GJB4-E204A was slightly lower in SDS-PAGE than GJB4-WT. As shown in Fig 3, GJA1 interacted with GJB4-E204A, but this was weaker than with GJB4-WT. These results suggest that GJB4-E204A may impair the formation of functional complexes consisting of both GJA1 and GJB4. We did not observe any change in the interaction of FLAG-GJB4-E204A with HA-GJB4-WT compared with the interaction of FLAG-GJB4-WT with HA-GJB4-WT (see S1 Fig). Furthermore, we did not observe any change in actin sarcomere organization in rat H9C2 cells overexpressing with GJB4-WT or GJB-E204A (see S2 Fig).

### GJB4 is expressed and colocalized with GJA1 in human diseased hearts

**Fig 4:**
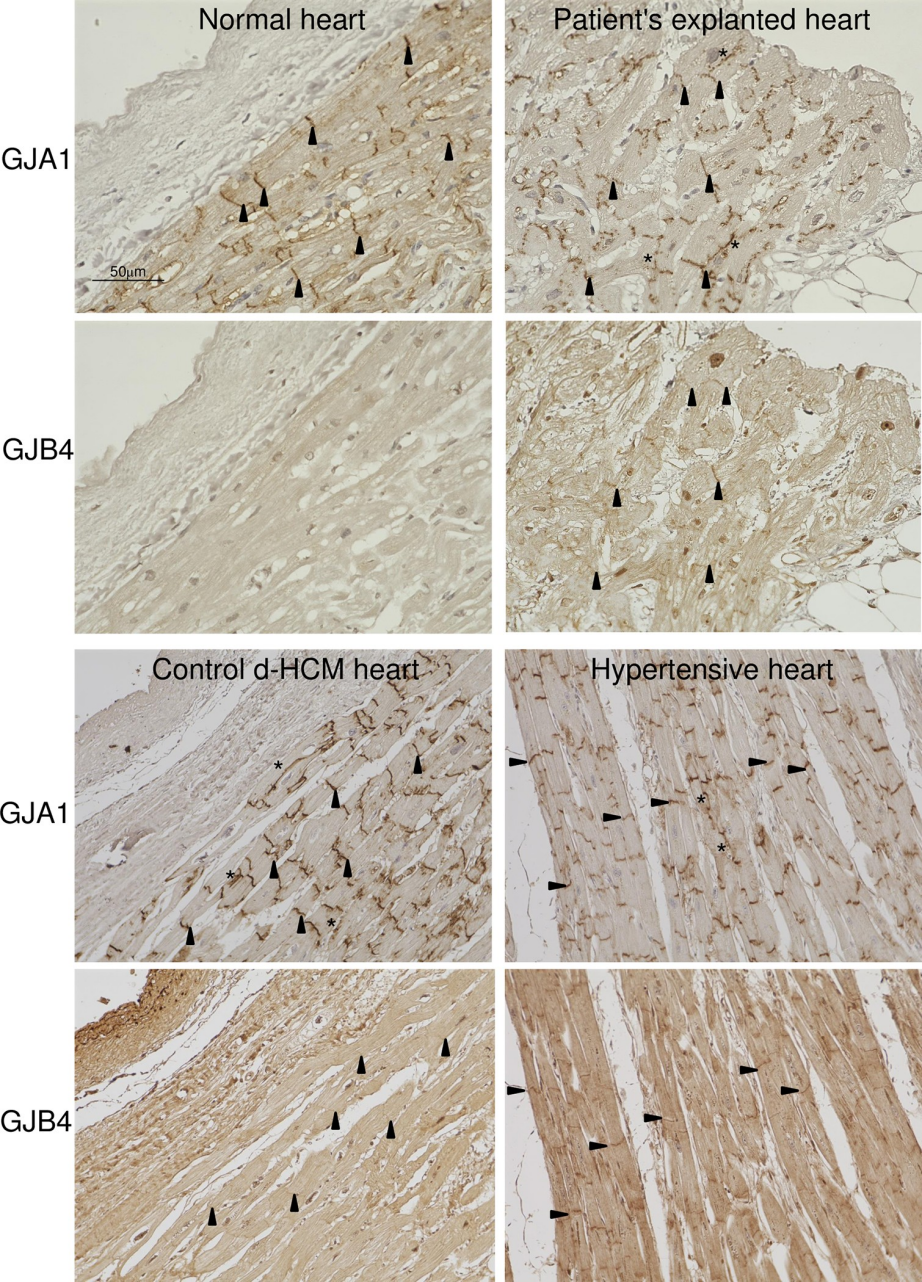
GJB4 is expressed and colocalized with GJA1 in human d-HCM and hypertensive hearts. GJA1 was expressed and localized at the intercalated disc (arrowheads) in autopsied normal (21 years old, male), control d-HCM (63 years old, male) and hypertensive hearts (73 years old, male), and the explanted heart from the present case, but GJB4 was expressed only in diseased hearts. Note the linear colocalization of GJA1 and GJB4 in the explanted heart. Lateralization (*) was observed partly for GJA1 expression but not for GJB4 expression.

We examined the localization of GJB4 in human diseased hearts and the explanted heart of the patient by immunohistochemical staining. GJB4 was colocalized with GJA1 in d-HCM and hypertensive hearts and in the heart of the present case, while GJB4 was never expressed in normal heart, as shown in rodent western blots (Figs 4 and 2). These results suggest that GJB4 plays a role in the remodeling of gap junctions when hearts are diseased. The expression of GJB4 in gap junctions was noted in the explanted heart compared with control d-HCM and hypertensive hearts (Fig 4). Lateralization, which is often recognized in diseased hearts, was observed only for GJA1 expression and not for GJB4 expression (Fig 4).

### GJB4 is increased and highly colocalized with GJA1 in GJB4-E204A iPSC-CMs

**Fig 5:**
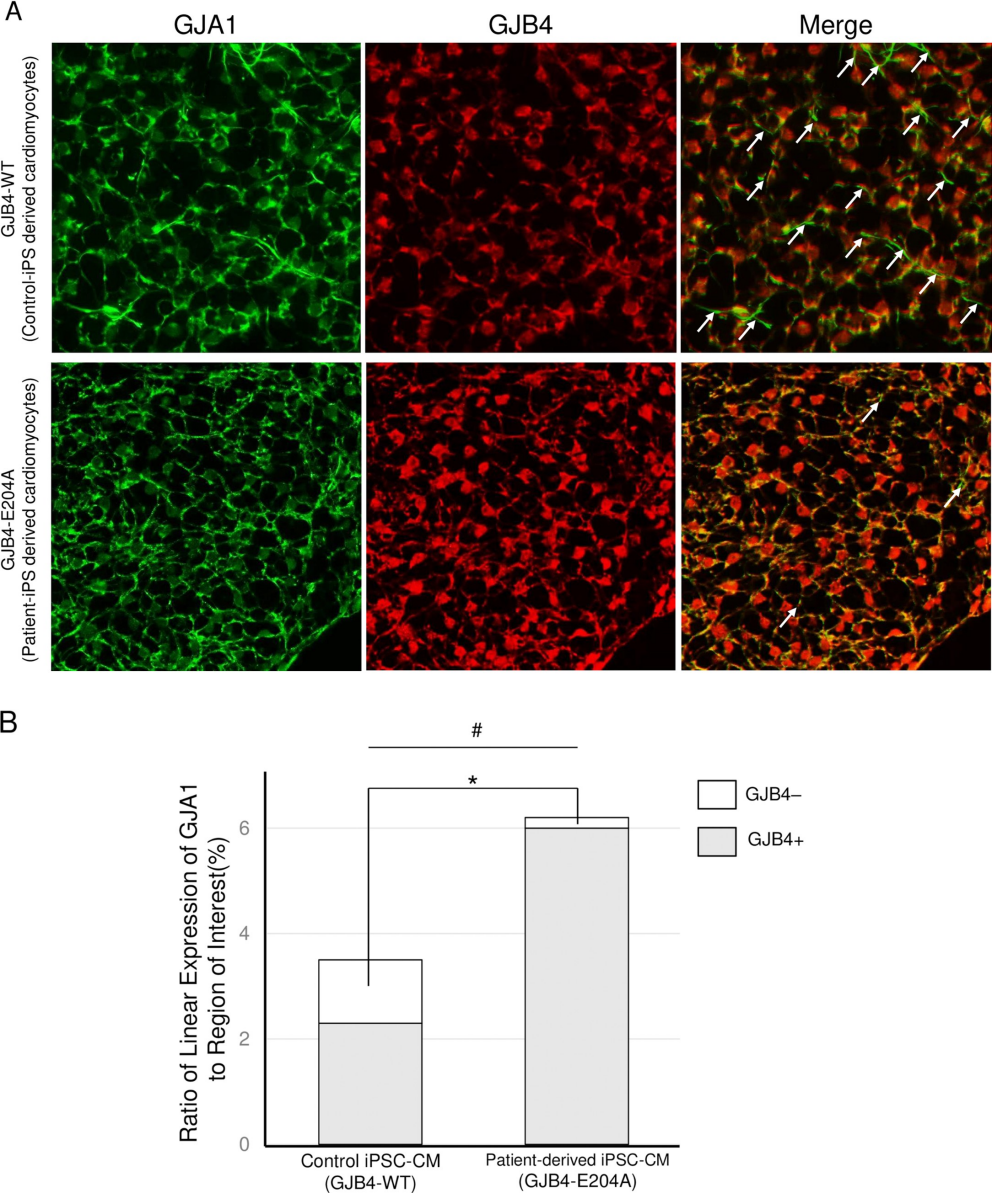
Colocalization of GJA1 and GJB4 in iPSC-induced cardiomyocyte spheres derived from control (GJB-WT) and the patient (GJB4-E204A). (A) GJA1 and GJB4 staining in cardiomyocytes spheres derived from iPSC-induced cardiomyocytes (CMs) of control and the patient. Arrows indicate the cellular boundaries of GJA- positive and GJB4-negative region. (B) Quantification of linear expression of GJA1 in cardiomyocytes spheres and percentages of GJB4-negative (GJB4–) and–positive (GJB4+) region. *P<0.05, the percentage of GJB4– in patient-derived iPSC- CM vs. in control. #P<0.05, the ratio of linear expression of GJA to region of interest in patient-derived iPSC-CM vs. control.

We developed patient’s specific iPSCs and examined the beating and the expression of GJB4 and GJA1 in iPSC-CM-derived spheres by a highly efficient differentiation technology. GJB4 was expressed in the cellular boundaries in addition to in the nucleus, while GJA1 was expressed only in the boundaries of cells (Fig 5). Although we did not observe any difference in the beating and the morphology of iPSC-CM spheres (S1 and S2 Videos), the expression of GJB4 was remarkably increased and colocalized with linear expression of GJA1 in patient’s iPSC-CM spheres compared with control cells (Fig 5). In control iPSC-CMs, there was two patterns of linear expression of GJA1. One was colocalized with GJB4 (GJB4+) and another was free of GJB4 (GJB4–). The estimated percentages of (GJB4–) was 34% in control iPSC-CMs (Fig 5B). However, we could observe only 3% of linear expression of GJA1 free of GJB4 in patient’s iPSC-derived spheres of cardiomyocytes. These results suggest the abnormal expression of GJB4, which should appear and disappear in appropriate timing of the redistribution of GJA1 in the development of beating iPSC-CMs spheres.

### GJB4-null zebrafish show impaired cardiac function

**Fig 6:**
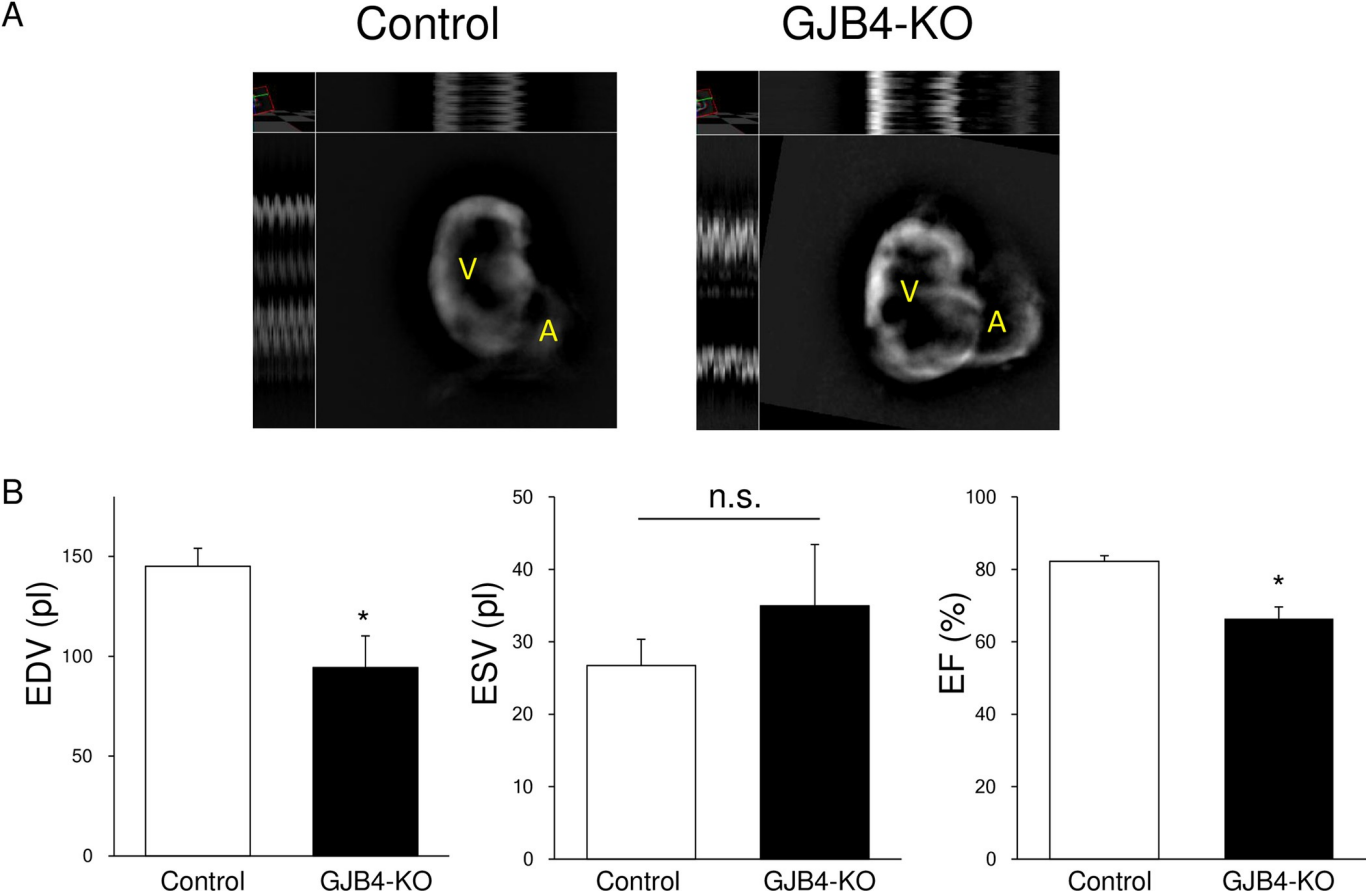
Increased endodiastolic volume and decreased ejection fraction in GJB4-deficient zebrafish. (A) Digitalized image of ventricles in control and GJB4-knockout zebrafish. V = ventricle, A = atrium. (B) Estimated endodiastolic volume (EDV) and endosystolic volume (ESV) and ejection fraction (EF) of ventricles in control and GJB4-knockout zebrafish. n = 12/group. *P<0.05 vs. control zebrafish. n.s. = no significance.

To examine the physiological role of GJB4 in the heart, we developed GJB4-null zebrafish by CRISPR/Cas9 and analyzed the volume and function of ventricles at 5 dpf (Fig 6 and S3 and S4 Videos). The EDV was significantly lower in GJB4-KO (94 pl, p<0.01) than in wild-type (WT, 145 pl) zebrafish at 5 dpf (Fig 6B, S3 and S4 Videos). Furthermore, the ventricular EF was decreased in GJB4-KO (0.66, p<0.01) compared with WT zebrafish (0.82), as shown in Fig 4B. These results indicate that GJB4 plays an important role in cardiac function in zebrafish.

## Discussion

Whole-exome sequencing via next-generation sequencing has been demonstrated to be able to detect rare disease-causing variants, especially in autosomal recessive forms of inherited diseases [20–22]. These newly identified genes of rare diseases can serve as new therapeutic targets and can be adapted from rare to more common diseases [23]. Therefore, using exome sequencing technologies for rare, inherited, and genetically heterogeneous forms of cardiovascular disease is very important. We identified 34 rare (≤ 0.10) variants with the use of whole exome sequencing in three participants with familial autosomal recessive d-HCM due to a marriage between cousins and proposed GJB4 as a candidate gene (Tables 1 and 2).

In human heart, it has been reported that GJA1, which has a half-life of 1.3 h [24], is the predominantly expressed isoform of GJP to form gap junctions and is normally located at the intercalated discs between cardiomyocytes. Recently, several lines of evidence have shown that alterations in GJA1 expression and distribution occur in hearts from patients with HCM, dilated cardiomyopathy (DCM), MI, and heart failure [7]. These alterations can be modified by several treatments including Ang II receptor blockers [25], calcium channel blockers [26], apocynin, an NADPH oxidase inhibitor [27], statins [28], and moderate intensity exercise [29]. In addition, GJA1-null mice die perinatally due to an obstruction of the right ventricular outflow tract of the heart [30]. Cardiomyocyte-specific deletion of GJA1 in mice causes sudden death due to slowed ventricular conduction within 2 months after birth [31]. Thus, GJA1 plays important roles in cardiac physiology, impulse propagation, hypertrophy, and function [32].

Although defects in GJB4 and GJA1 in humans are known to cause hearing loss [33, 34], GJB4 can be expressed in immature cardiomyocytes [8]. Importantly, GJB4 can work as a normal functional channel like GJA1 [5]. To the best of our knowledge, this is the first report to show that GJB4 is induced in diseased hearts in humans, rats, and mice (Figs 2, 4 and 5). Interestingly GJB4 was not detected in any hearts from WT or sham models of rats and mice (Fig 2). These results indicate that GJB4 can be induced to constitute gap junctions only when a heart is burdened.

It has been reported that gap junctions are remodeled by changing the distribution and expression of GJP under cardiac hypertrophy and fibrosis [32]. It has also been reported that an increase and a reduction in the expression of GJA1 occurs in compensated and decompensated hypertrophy, respectively [7]. Indeed, reductions in the expression and heterogeneous redistribution of GJA1 have been reported in decompensated human hypertrophy [35], arrhythmogenic cardiomyopathy [36], ischemic samples [35], and induced -pluripotent stem cell (iPS)-derived cardiomyocytes from patients with cardiomyopathy [37]. However the density of intercalated discs does not seem to change in chronic hypertrophied or ischemic myocardium [35]. Therefore, it is possible that intercalated discs continue to exist with less GJP, or that other kinds of GJP are expressed to replace GJA1. Indeed, it has been proposed that GJA5 can be increased to compensate for the loss of GJA1 in ischemic cardiomyopathy and DCM [38]. We observed the induction of GJB4 in rodent diseased hearts, and it colocalized with GJA1 in human diseased hearts. These results indicate that GJB4 can interact with GJA1 in gap junctions and play some roles in cardiac remodeling. GJB4-E204A failed to interact with GJA1 effectively compared with GJB4-WT (Fig 3). Interestingly, the expression of GJB4 was exaggerated in the explanted heart of the GJB4-E204A patient compared with control d-HCM hearts (Fig 5). This result suggests that the heart of the present case needs more GJB4 to compensate for GJA1 and reconstitute the gap junctions, because GJB4-E204A does not interact with GJA1 effectively. Indeed more GJA1 was induced to interact with GJB4-E204A in iPSC-CMs spheres (Fig 5). This fact may suggest excess GJA1-GJB4 complex induce hypertrophic and arrhythmogenic condition. Indeed abnormal expression of GJP complex is often associated with abnormal conduction and arrhythmia [7]. On the other hand, GJB4-deficient zebrafish showed impaired systolic function with partial dyskinetic motion in ventricle walls (Fig 6 and S3 and S4 Videos), suggesting that GJB4 is required for normal cardiac structure and function. Further studies are necessary to reveal the complete roles of GJB4 and the prevalence and effects of GJB4 mutant in cardiac diseases in humans.

It has been reported that the lateralization of GJA1 occurs in cardiomyocytes of compensated cardiac hypertrophy in patients with aortic stenosis [39]. GJA1 is originally localized at the intercalated discs but moves away from them to the lateral membranes following hypoxia and cellular stress, leading to limited cell-cell connectivity [40]. In contrast to GJA1, we did not observe any lateralization or localization of GJB4 in other portions of hearts, except for in the transverse boundaries of myocytes, i.e. intercalated discs (Fig 4).

In conclusion, our findings suggest that GJB4 is a new GJP in human heart and plays important roles in cardiac function and remodeling, and that a GJB4 mutant may cause familial autosomal recessive HCM, which can progress into severe d-HCM. GJB4 is induced to compensate for GJA1 in heart only when a heart is burdened in humans, rats, and mice. Therefore, GJB4 may be an important therapeutic target for preventing the development of cardiac hypertrophy and heart failure. However, further studies are required to determine how GJB4 mutations lead to the dysregulation of the GJP complex and how GJB4 affects the transition from cardiac hypertrophy and arrhythmia to heart failure.

### Study limitations

We cannot rule out the possibility that an alternative exon, intron, or non-coding RNA is the cause of this familial HCM because we could perform only known exome analysis. In addition, we cannot rule out the possibility that the patient’s father and/or mother does not show the phenotype of HCM but has a homozygous gene mutation.

## Supporting information

S1 Fig Similar interaction of GJB4-E204A with GJB4-WT to GJB4-WT. Coimmunoprecipitation of GJB4-E204A and GJB4-WT in Cos7 cells overexpressing FLAG-GJB4-E204A or FLAG-GJB4-WT and HA-GJB4-WT (n = 3).
(PPTX)

S2 Fig No effect of GJB4-E204A on sarcomere organization. H9C2 cells overexpressing GJA1 and GJB4-WT or GJB4-E204A were stained with rhodamine-phalloidin (n = 3).
(PPTX)

S1 Table Summary of whole exome sequencing analysis. (DOCX)

S2 Table Nucleotide sequences of crRNA, tracrRNA, and PCR primers used for this study. (DOCX)

S1 Video (MP4)

S2 Video (MP4)

S3 Video (MP4)

S4 Video (MP4)

S1 File (ZIP)

## Abbreviations

ADM: adriamycin
CRT-D: cardiac resynchronization therapy-defibrillator
Cx: connexin
DCM: dilated cardiomyopathy
GJA1 (Cx43: gap junction protein A1
GJA5 (Cx40): gap junction protein A5
GJB4 (Cx30.3): gap junction protein beta 4
GJC1 (Cx45): gap junction protein gamma 1
GJP: gap junction protein
HCM: hypertrophic cardiomyopathy
iPSC: induced pluripotent stem cell
MI: myocardial infarction
PAH: pulmonary arterial hypertension
PAF: paroxysmal atrial fibrillation
SNP: single nucleotide polymorphism
WT: wild-type
