# Supplementary figures and images for: Gap junction protein beta 4 plays an important role in cardiac function in humans, rodents, and zebrafish
Source: PLoS One. 2020 Oct 13;15(10):e0240129. doi: 10.1371/journal.pone.0240129 (PMC7553298; doi:10.1371/journal.pone.0240129)

## Slide 1
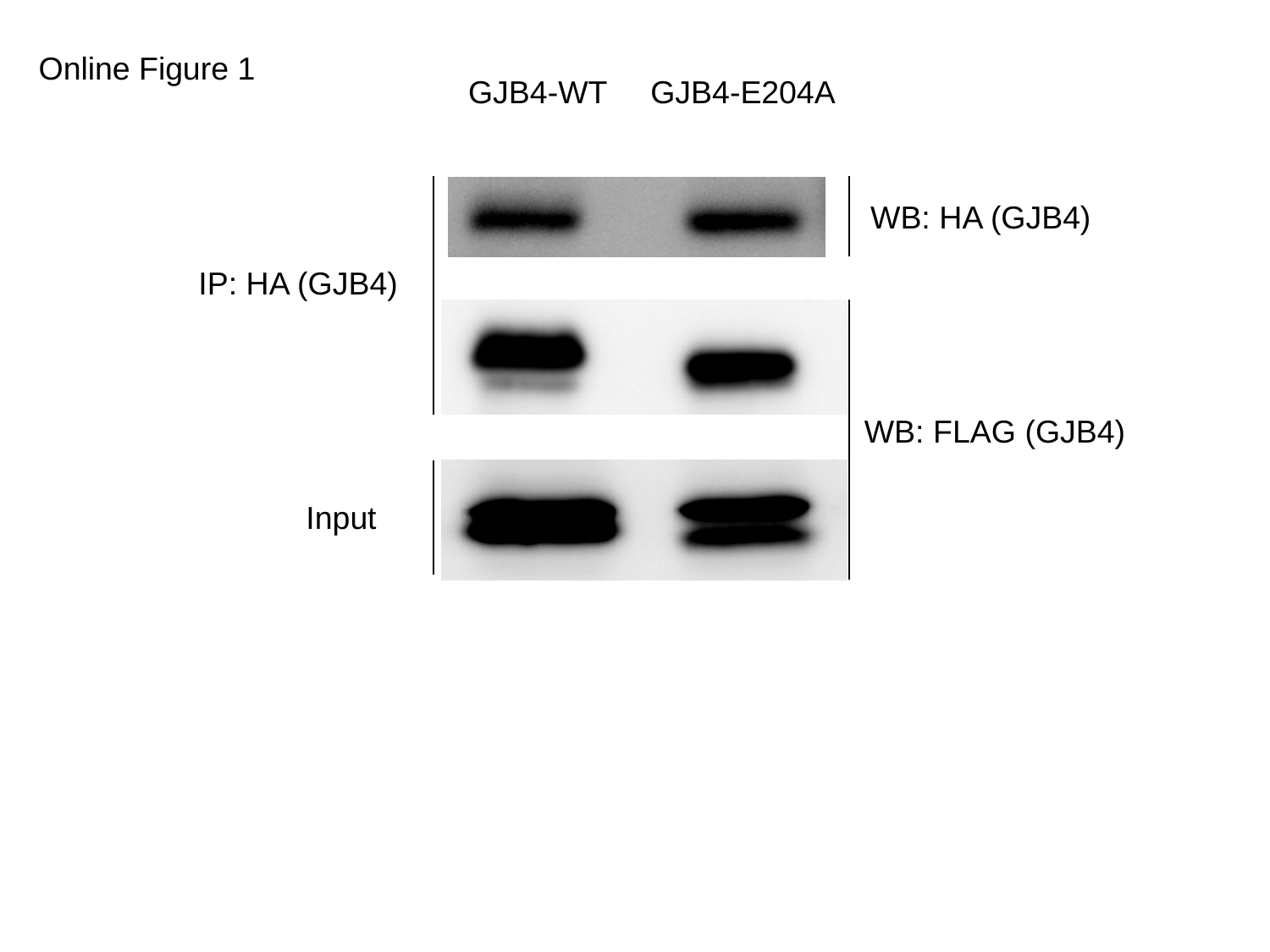

Online Figure 1
GJB4-WT
GJB4-E204A
WB: HA (GJB4)
IP: HA (GJB4)
WB: FLAG (GJB4)
Input

Supplement: S1 Fig — Coimmunoprecipitation of GJB4-E204A and GJB4-WT in Cos7 cells overexpressing FLAG-GJB4-E204A or FLAG-GJB4-WT and HA-GJB4-WT (n = 3). (PPTX) [file pone.0240129.s001.pptx]
