# Supplementary material for: Gap junction protein beta 4 plays an important role in cardiac function in humans, rodents, and zebrafish
Source: PLoS One. 2020 Oct 13;15(10):e0240129. doi: 10.1371/journal.pone.0240129 (PMC7553298; doi:10.1371/journal.pone.0240129)
Supplement: S2 Fig — H9C2 cells overexpressing GJA1 and GJB4-WT or GJB4-E204A were stained with rhodamine-phalloidin (n = 3). (PPTX) [file pone.0240129.s002.pptx]

## Slide 1
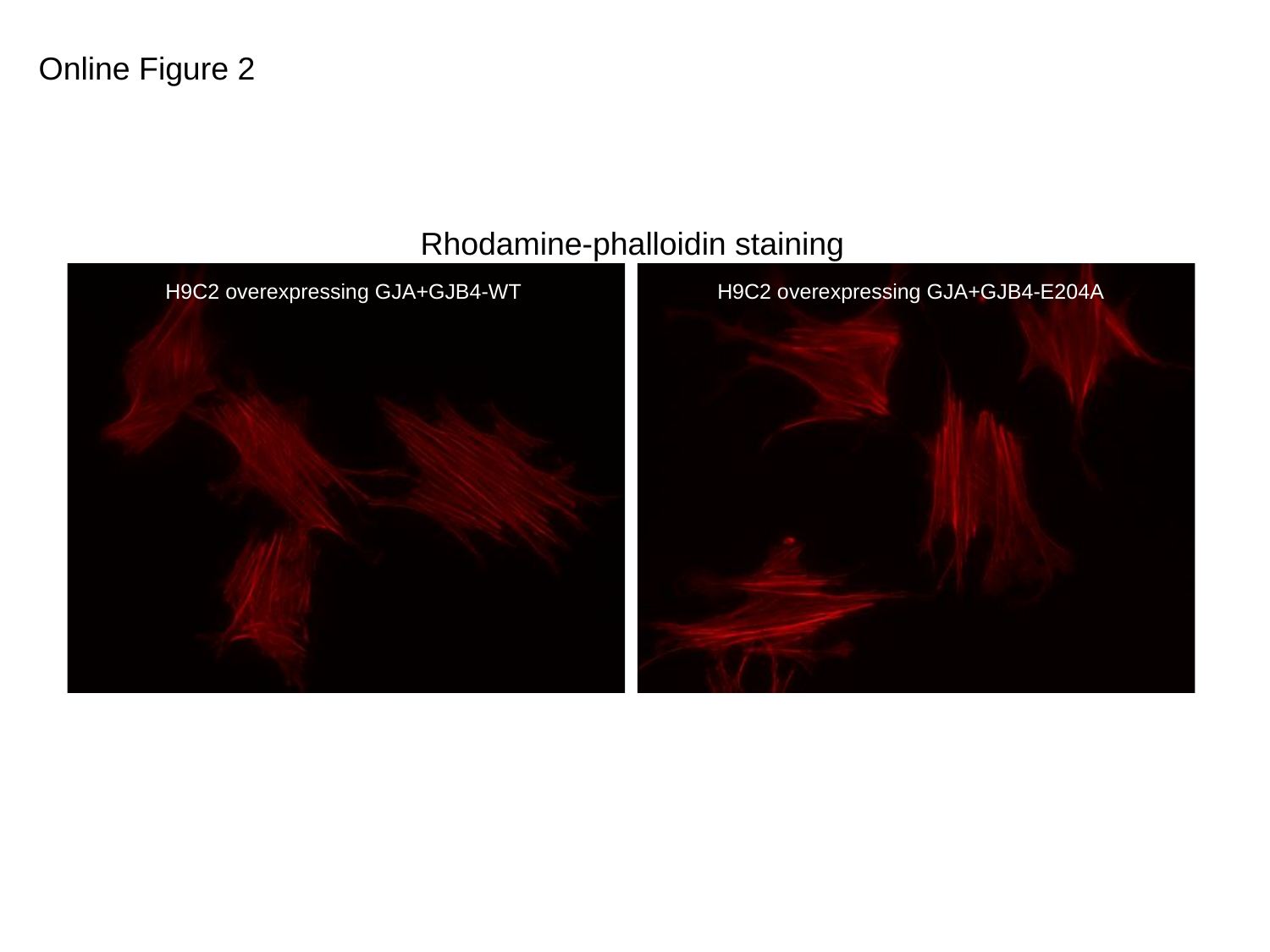

Online Figure 2
Rhodamine-phalloidin staining
H9C2 overexpressing GJA+GJB4-WT
H9C2 overexpressing GJA+GJB4-E204A
