# Supplementary material for: Gap junction protein beta 4 plays an important role in cardiac function in humans, rodents, and zebrafish
Source: PLoS One. 2020 Oct 13;15(10):e0240129. doi: 10.1371/journal.pone.0240129 (PMC7553298; doi:10.1371/journal.pone.0240129)
Supplement: S1 Table — (DOCX) [file pone.0240129.s003.docx]

**S1 Table. Summary of whole exome sequencing analysis**

| Items | Mother | Father | Patient |
| --- | --- | --- | --- |
| Total sequencing yields (Gbp) | 12.8 | 12.2 | 8.7 |
| % Mappable reads (/total reads) | 92.76 | 91.33 | 90.53 |
| % Coverage of target regions (more than 20X) | 94.38 | 92.57 | 83.36 |
| Mean read depth of target regions | 201.9 | 189.1 | 133.3 |
