# Supplementary material for: Gap junction protein beta 4 plays an important role in cardiac function in humans, rodents, and zebrafish
Source: PLoS One. 2020 Oct 13;15(10):e0240129. doi: 10.1371/journal.pone.0240129 (PMC7553298; doi:10.1371/journal.pone.0240129)
Supplement: S2 Table — (DOCX) [file pone.0240129.s004.docx]

**S2 Table.** Nucleotide sequences of crRNA, tracrRNA, and PCR primers used for this study

| name | sequence (5'-3') |
| --- | --- |
| Cx30.3_crRNA | agucgcggcugaaaaaguguguuuuagagcuaugcuguuuug |
| tracRNA | aaacagcauagcaaguuaaaauaaggcuaguccguuaucaacuugaaaaaguggcaccgagucggugcu |
| Cx30.3_gF1 | ccgtgtctggctctcgatag |
| Cx30.3_gR1 | gatgagttgcagagcccaga |
